# Supplementary material for: Fluid movements enhance creative fluency: A replication of Slepian and Ambady (2012)
Source: PLoS One. 2020 Jul 30;15(7):e0236825. doi: 10.1371/journal.pone.0236825 (PMC7392226; doi:10.1371/journal.pone.0236825)
Supplement: S1 File — (PDF) [file pone.0236825.s001.pdf]

Supplementary results including all participants

In the main text, to eliminate the potential effects of laterality on motor properties, we reported results excluding three participants who used their left hand in the tracing task. Tables S1 and S2 summarize the results including all 63 participants. The analytic procedures were the same as those described in the main text. Findings were comparable between the reduced and full samples. Participants in the fluid condition generated a larger number of alternative uses for newspapers than did those in the non-fluid condition, regardless of mood, subjective difficulty, and newspaper subscription. There was no group difference in originality.

Table S1. Descriptive statistics and group differences including all participants.

	Fluid condition, $n = 31$	Non-fluid condition, $n = 32$	Test of group difference
Age	17.2 (0.9)	17.3 (0.7)	$t(61) = 0.28$, $p = 0.780$, $d = -0.07$ [-0.46, 0.35]
Left-hand use (*n*)	1	2	$\chi^2(1) = 0.32$, $p = 0.573$, $V = 0.07$
Fluency	4.13 (1.36)	3.38 (1.39)	$t(61) = 2.18$, $p = 0.033$, $d = 0.55$ [0.06, 1.45]
Originality	2.85 (0.52)	2.69 (0.57)	$t(61) = 1.13$, $p = 0.264$, $d = 0.28$ [-0.12, 0.43]
Mood rating	5.94 (1.90)	5.56 (1.22)	$t(50.9) = 0.93$, $p = 0.359$, $d = 0.24$ [-0.44, 1.18]
Difficulty rating	4.00 (2.11)	4.19 (2.29)	$t(61) = -0.34$, $p = 0.737$, $d = -0.09$ [-1.30, 0.92]
Subscription (*n*)	22	24	$\chi^2(1) = 0.13$, $p = 0.718$, $V = 0.05$

Values indicate mean (SD in parentheses) except for the number of left-handers and subscribers; 95% confidence interval in square brackets.

Table S2. Generalized linear models including all participants.

	Fluency [a]			Originality [b]		
	Estimate	*z*	*p*	Estimate	*z*	*p*
Intercept	1.33 [1.21, 1.43]	23.85	< 0.001	1.02 [0.96, 1.07]	35.27	< 0.001
Condition	-0.24 [-0.48, -0.03]	-2.16	0.035	-0.09 [-0.20, 0.02]	-1.53	0.132
Mood rating	0.00 [-0.08, 0.09]	0.05	0.962	0.01 [-0.03, 0.04]	0.31	0.754
Difficulty rating	0.02 [-0.03, 0.06]	0.74	0.462	-0.01 [-0.03, 0.02]	-0.64	0.524
Subscription	-0.05 [-0.27, 0.19]	-0.44	0.661	0.00 [-0.12, 0.12]	0.01	0.996
Condition*Mood	0.03 [-0.13, 0.20]	0.44	0.662	0.02 [-0.06, 0.09]	0.46	0.647
Condition*Difficulty	0.03 [-0.06, 0.12]	0.63	0.534	-0.04 [-0.09, 0.01]	-1.64	0.108
Condition*Subscription	0.17 [-0.28, 0.64]	0.74	0.465	0.14 [-0.09, 0.39]	1.19	0.238

[a] $R^2 = 0.107$. [b] $R^2 = 0.127$. Asterisks indicate interaction; 95% confidence interval in square brackets.
