# Supplementary material for: Fluid movements enhance creative fluency: A replication of Slepian and Ambady (2012)
Source: PLoS One. 2020 Jul 30;15(7):e0236825. doi: 10.1371/journal.pone.0236825 (PMC7392226; doi:10.1371/journal.pone.0236825)
Supplement: Attachment — Submitted filename: authorResponse.docx [file pone.0236825.s003.docx]

We thank the Editor and Reviewers for their favorable evaluations and helpful comments on our manuscript. Please find our responses (black font) to each comment.

**Editor**

Since literature about embodied creative cognition is not so broad, I suggest to take into account and mention all the few studies which have been conducted on such a topic. So, I suggest these further papers, beside those reported by Reviewer 3:

Andolfi, V. R., Di Nuzzo, C., & Antonietti, A. (2017). Opening the mind through the body: The effects of posture on creative processes. Thinking Skills and Creativity, 24, 20-28. doi: 10.1016/j.tsc.2017.02.01

Friedman, R. S., Fishbach, A., Förster, J., & Werth, L. (2003). Attentional priming effects on creativity. Creativity Research Journal, 15, 277–286. doi:10.1080/10400419.2003.9651420

Ijzerman, H., Leung, A. K.-Y., & Ong, L. (2014). Perceptual symbols of creativity: Coldness elicits referential, warmth elicits relational creativity. Acta Psychologica, 148, 136–147. doi:10.2139/ssrn.2235561

(I exclude papers about the effects of physical activity on creativity, which is an issue not so central to what the authors addressed in the paper).

We integrated the papers you proposed [15-17] into the Introduction as follows:

Line 47: Recent studies have suggested that somatosensory inputs can activate such conceptual metaphorical mapping and simultaneously or subsequently affect creative thinking. For example, since openness is metaphorically associated with divergence in creative thinking, open body postures lead to better divergent creative performance than closed postures [15]. Similarly, activities involving larger areas of the facial muscles lead to broader perceptual and conceptual attention, facilitating the originality of generated ideas [16]. Furthermore, physical warmth and coldness foster relational and referential creativity, respectively [17].

**Reviewer #1**

The only change I would suggest is how the authors discuss Thomas & Lleras, 2009 (reference 11). Here, the authors write, "For example, insight problem solving and divergent thinking are facilitated by swinging compared to stretching arm movements [11]" but this does not capture the reason why. In the specific study the authors cite here, participants were tasked with completing a specific insight puzzle: Maier's two-string problem. To solve the puzzle, the participant has to recognize that the string can be used to make a *swinging* pendulum. That's the key insight to solving the puzzle, and when participants practice this movement, they were able to figure it out more quickly. In this study, the shape of the movement helped because it physically matched an element of the solution.

Thank you for pointing out our misinterpretation. We revised to cite Thomas & Lleras [14] as the study showing the effect of the shape of movement trajectory on problem solving, as follows:

Line 39: Moreover, the shape of the trajectory of bodily movement can provide an implicit clue to insight problem solving; for instance, swinging the arm facilitates solving problems about making a swinging pendulum with a string [14].

**Reviewer #2**

Sometimes concision hinders clarity, for instance I would recommend to explain more the studies reported in lines 41-43

Thank you for your helpful comments. We removed these studies from the manuscript according to a comment by Reviewer #3.

Lines 85-86: it is not clear to me why authors state that it is still unsure, based on the original study, whether mood and task difficulty impacted fluidity and originality. It should be better specified in what respect the original paper missed the point (as in it previously said that Slepian and Ambady already considered these possible confounding variables)

Our previous manuscript was misleading. Since Slepian and Ambady (2012) showed no difference in mood and subjective difficulty between fluid and non-fluid conditions, it remains unclear whether mood and subjective difficulty affect fluency and originality, and whether mood and subjective difficulty interact with the effect of fluidity. To clarify these points, we revised relevant parts in the manuscript. Please see below:

Line 84: Slepian and Ambady [23] confirmed that self-reported mood (Experiment 1) and the subjective difficulty of tracing line-drawings (Pilot Experiment) did not differ between fluid and non-fluid arm movements. However, it remains unclear whether mood and subjective difficulty during the tracing task affect the fluency and originality, and whether they interact with the effects of fluidity.

L 95: Third, as mentioned above, whether mood and subjective difficulty affect divergent creative thinking and interact with the effects of fluidity should be examined.

Figure 1 is very “old style” and inconsistent with the strong statistical analyses conducted. It could be replaced by a jittered plot that is more informative about the sample distribution (Jamovi creates nice ones!)

We uploaded a new Figure 1 with jittered individual data and error bars which indicate 95% confidence interval. We also revised Fig 1 legend as follows:

L 192: **Fig 1. Results of the alternative uses task.** (A) Fluency and (B) originality scores for the fluid and non-fluid conditions. Dots represent individual data points. Error bars denote 95% confidence interval.

I would move the lines 212-217 and Table 3 to the results section. It is not usual to see such results details in the discussion. In the discussion it is worth commenting on the meaning of the differences found between cultures.

The analysis you pointed out was moved to the Results section. Accordingly we also revised the Discussion section. Please see below:

L 207: **Follow-up analysis**

The original study [23] reported larger effect sizes of fluidity on fluency and originality (ds = 1.04, 0.87, respectively) than did the present study (ds = 0.60, 0.30, respectively). To explore possible reasons for these discrepancies, we compared fluency and originality scores in both conditions between the original and present studies. Table 3 summarizes the results from Experiment 1 of the original study [23]. Since the results were only visually reported, we extracted the values from the figure using a digitizer (WebPlotDigitizer [36]). The effect sizes of the differences between the original and present studies (dstudy) were calculated [37]. Substantially large values of dstudy suggest that participants in the original study performed better than did those in the present study, except for originality in the non-fluid condition.

L 250: The size of the significant effect of fluidity on fluency was smaller in this study than in the original study [23]. Moreover, the original study found a significant effect of fluidity on originality, while the present study did not. To explore the factors underlying these discrepancies, our follow-up analysis compared fluency and originality scores between the original and present studies and showed large values of dstudy (except for originality in the non-fluid condition), suggesting that participants in the original study showed higher fluency and originality scores. Given that the original study employed American undergraduates, cultural differences could have affected divergent creative thinking.

**Reviewer #3**

Abstract: Line 10, I recommend to the authors to be less general and to make more explicit the statement.

Thank you for your helpful comments. We revised to make the sentence less general.

Line 10: Bodily movements representing abstract concepts (e.g., fluidity) can affect divergent creative thinking.

Introduction:

Lines 24-25 : I would consider also the fact the multiple representation views of Abstract Concept. Abstract concepts involve also linguistic, emotional and social experiences as well as internal experiences. See: Borghi, A. M., Barca, L., Binkofski, F., & Tummolini, L. (2018). Varieties of abstract concepts: development, use and representation in the brain. Philosophical Transactions of the Royal Society B: Biological Sciences, 373(1752).

Borghi, A. M., & Binkofski, F. (2014). Words as social tools: An embodied view on abstract concepts (Vol. 2). Springer New York.

I would not neglect the social component of abstract concepts as well, compared to the concrete one. See Fini, C., & Borghi, A. M. (2019). Sociality to Reach Objects and to Catch Meaning. Frontiers in psychology, 10.

At Line 24, we described that “Abstract concepts are grounded in language, emotions, and sociality [1,2]” while citing Borghi et al. (2018) and Fini & Borghi (2019).

Lines 29-30: Please specify that you are talking about abstract concepts

Done (Line 29-31).

Could you please better clarify and make explicit the theoretical shift from the fact the bodily movements affect insight problem solving and divergent thinking to the fact that concepts represented by bodily movements can affect creativity. The passage has to be smooth for the reader.

We revised the passages according to the reviewer’s suggestion. Note that, based also on the Editor’s comment, we increased these paragraph while newly citing some papers.

Line 36: Studies on embodied cognition have also revealed that bodily sensation and movements can modulate divergent creative thinking [11, 12]. For example, walking better facilitates subsequent divergent thinking than sitting [13], suggesting an effect of bodily movements per se on creativity. Moreover, the shape of the trajectory of bodily movement can provide an implicit clue to insight problem solving; for instance, swinging the arm facilitates solving problems about making a swinging pendulum with a string [14]. These findings suggest that physical components of bodily movements facilitate divergent creative thinking and problem solving.

Abstract concepts represented by somatosensation and bodily movements also modulate creativity [11, 12]. To understand abstract concepts, human cognitive as well as linguistic processes employ metaphors mapping abstract concepts onto superficially dissimilar concrete concepts [3, 5]. Recent studies have suggested that somatosensory inputs can activate such conceptual metaphorical mapping and simultaneously or subsequently affect creative thinking. For example, since openness is metaphorically associated with divergence in creative thinking, open body postures lead to better divergent creative performance than closed postures [15]. Similarly, activities involving larger areas of the facial muscles lead to broader perceptual and conceptual attention, facilitating the originality of generated ideas [16]. Furthermore, physical warmth and coldness foster relational and referential creativity, respectively [17]. More importantly for the present study, bodily movements enacting conceptual metaphors can also affect creativity. For example, the metaphorical phrase “think outside the box” refers to thinking about something regardless of given rules and constraints. Bodily movements enacting this metaphor, such as thinking while sitting outside (or inside) a box and walking freely (or alongside a path), can enhance divergent and convergent creative thinking [18].

Lines 42-43 I do not agree with the fact that arm flexion movements represents an approaching behavior: see De Houwer, Crombez, Baeyens, & Hermans, 2001, Krieglmeyer, De Houwer, & Deutsch, 2011, Markman & Brendl, 2005, Seibt, Neumann, Nussinson, & Strack, 2008; Schneirla, 1959 according to which positive/negative stimuli lead to approach/avoidance tendencies understood as tendencies to decrease/increase distance irrespective of the specific muscle movements involved.

According to the reviewer, we removed the sentences referring to the flexion-movement papers.

Line 49-50 Too nebulous these sentences

We revised to make this paragraph clearer.

L 59: It is theorized that human intelligence consists of fluid and crystallized aspects [19, 20]. While crystallized intelligence refers to acquired knowledge, fluid intelligence includes flexibility and fluency of thought. On the other hand, the ability to fluently generate ideas is referred to as divergent thinking and is the basis of creativity [21]. Since a physical fluid moves divergently, divergent thinking is conceptually or metaphorically associated with fluidity [22]. This association might be represented in cognitive processes, and if fluidity is enacted by bodily movements depicting a fluid trajectory, they might also activate divergent creative thinking.

Line 54 Please specify what you mean for “fluid movements” already

We revised to say “bodily movements depicting a fluid trajectory” at Line 65.

Line 61 “The generation of many alternative uses of newspapers” is a neuropsychological task which can be considered under which label?

This is the alternative uses task, which has been employed in cognitive psychology (Guilford, 1967) [ref. 21] not limited to neuropsychology. The sample object in this task is not limited to newspapers. Previous studies also used other daily objects such as brick, paperclip, scissors, and so on.

Statistical analysis:

I am not familiar with the method implemented by the authors to analyze to predict the fluency and the originality scores. Can you better explain it? Clarify that you have a fluency dependent variable and a Fluency predictor variable, it is not clear.

The fluency and originality scores were dependent variables. Our generalized linear model was fitted separately to the fluency and the originality. For details, please see below:

L 172: To check the interactive effects of the fluidity of arm movements and the other variables on the fluency and originality, we fitted a generalized linear model with a log link function and a Gaussian distribution. The exponential family distribution was determined based on the minimization of the Akaike information criterion. The predicted variables were the fluency and originality scores. The predictor variables were the condition (fluid or non-fluid), mood score, difficulty score, and newspaper subscription (yes or no) in addition to interactions of the condition with mood, difficulty, and subscription.

Why did you not implement e backward stepwise procedure to compare different models and choose the best in the goodness of fit? I mean why did you not perform a Likelihood ratio test to compare different models varied for the complexity of the random and the fixed effects structures?

We used the generalized linear model with log link function and Gaussian distribution, because this was the best fitted model for our data according to the minimization of Akaike information criterion (AIC). We believe that, when we rely on the AIC, likelihood-ratio test is not necessary.

Results:

Can you report exactly the means of the Fluency score?

In the Scoring Creativity section, we have already reported how to calculate the fluency score. Nevertheless, we revised it for more clarification. Please note that, to avoid redundancy, we decided not to report the same in Results section.

L 151: The uses reported by each participant were coded as fluency and originality according to the original study [23]. Each participant’s fluency score was defined as the number of alternative uses for newspapers reported within one minute.

Discussion:

Please speculate more on the link about the bodily fluency and the conceptual fluency.

We added a new paragraph (see below) to discuss a selective effect of fluidity on fluency and a link between fluency in arm movement and divergent thinking.

L 233: Our results showed a selective effect of fluid arm movements on creative fluency, suggesting a link between fluency in manual movements and creative generation in metaphorical and cognitive processes. There are several domains of divergent creative thinking, such as fluency, originality, and flexibility [38]. We speculate that it is possible that a certain abstract concept selectively relates to and activates a particular aspect of divergent thinking. Indeed, another selective effect of arm movements was suggested. In a recent study [39], participants who performed arm movements tracing out large circles generated more original ideas (i.e., originality) than those who traced out small ones, but the total number of generated ideas (i.e., fluency) was comparable between conditions, suggesting that the largeness or divergence represented by arm movements could selectively influence originality. Therefore, kinematic properties such as curvature and size may determine the aspect of divergent creative thinking that is affected. We cannot rule out the possibility that the size of the drawings in our task was insufficient to affect originality.

Could you please enrich the discussion with relevant embodied literature?

I also recommend to enrich the embodied literature in these papers:

Era, V., Boukarras, S., & Candidi, M. (2019). Neural correlates of action monitoring and mutual adaptation during interpersonal motor coordination: Comment on" The body talks: Sensorimotor communication and its brain and kinematic signatures" by G. Pezzulo et al. Physics of life reviews, 28, 43.

Kuo, C. Y., & Yeh, Y. Y. (2016). Sensorimotor-conceptual integration in free walking enhances divergent thinking for young and older adults. Frontiers in psychology, 7, 1580.

We integrated papers you suggested (Kuo & Yeh, 2016; Era et al., 2019) [40, 41] into the Discussion section as follows:

L 246: A recent study has suggested that the effect of fluid movements on divergent creative thinking may be generalized to whole-body movements (i.e., walking) [40]. Participants who walked freely rather than along a rectangular path showed greater fluency and originality scores. The comparison between free and rectangular walks may also serve as a comparison between fluid and non-fluid movements. If so, the null effect of fluidity on originality that we found could be attributable to the difference between motor effectors (i.e., manual drawing versus whole-body walking). To speculate, fluidity could be represented more effectively by richer sensorimotor information conveyed through whole-body movements, consequently affecting divergent thinking more strongly. A direct comparison between motor effectors is needed in future studies. Furthermore, from the viewpoint of social and applied contexts, interaction with and observation of others’ movements could also provide sensorimotor information that facilitates creativity, because the sensorimotor system in the brain is able to integrate observed and executed bodily movements, facilitating motor planning and execution [41]. We may speculate that joint action (e.g., line-drawing synchronously performed by individuals face to face) could enhance the effect of metaphorical movements on creativity and open a new avenue for research on embodied creativity.
